# A Review on the Pathogenesis and Clinical Management of Placental Site Trophoblastic Tumors

**DOI:** 10.3389/fonc.2019.00937

**Published:** 2019-11-28

**Authors:** Xuan Feng, Zhi Wei, Sai Zhang, Yan Du, Hongbo Zhao

**Affiliations:** ^1^Obstetrics and Gynecology Hospital, Fudan University, Shanghai, China; ^2^Department of Obstetrics and Gynecology, Shanghai Medical School, Fudan University, Shanghai, China; ^3^Shanghai Key Laboratory of Female Reproductive Endocrine Related Diseases, Shanghai, China

**Keywords:** placenta site trophoblastic tumor, clinical presentation, mechanism, treatment, prognosis

## Abstract

Placental site trophoblastic tumor (PSTT) is a rare type of gestational trophoblastic disease originating from the intermediate trophoblast. Compared with hydatidiform mole, invasive hydatidiform mole and choriocarcinoma, the diagnosis of PSTT is more complicated and lacks specific and sensitive tumor markers. Most PSTT patients demonstrate malignant potential, and the primary treatment of PSTT is hysterectomy. However, metastasis occasionally occurs and even causes death in a small number of PSTT patients. Most PSTT patients are young women hence fertility preservation is an important consideration. The major obstacle for PSTT patient prognosis is chemotherapy resistance. However, the current understanding of the pathogenesis of PSTT and clinical treatment remains elusive. In this review, we summarized the research progress of PSTT in recent years from three aspects: mechanism, clinical presentation, and treatment and prognosis. Well-conducted multi-center studies with sufficient sample sizes are of great importance to better examine the pathological progress and evaluate the prognosis of PSTT patients, so as to develop prevention and early detection programs, as well as novel treatment strategies, and finally improve prognosis for PSTT patients.

## Introduction

Placental site trophoblastic tumor (PSTT) is a rare type of gestational trophoblastic disease (GTD) originating from the placental implantation site. It was first named as “trophoblastic pseudotumor” by Kurman and Scully in 1976 (1). With more understanding of this disease, the terminology of “PSTT” has gradually been acknowledged to feature both the benign and malignant potentials of this specific type of tumor. The World Health Organization (WHO) listed PSTT as the fourth trophoblastic disease in 1944, along with hydatidiform mole, invasive hydatidiform mole, and choriocarcinoma. Among these tumors, partial or complete hydatidiform mole is considered to be benign hyperplasia of the villi, while choriocarcinoma and PSTT are considered as tumor tissues (2). A global survey showed that the disease-specific mortality of PSTT is higher than other GTD subtypes (16.1% for PSTT, 6.5% for hydatidimolars, and 13.4% for choriocarcinoma) (3). The characteristics of PSTT lies in its unpredictable biological behavior, poor prognosis compared with other subtypes of GTD, and less responsiveness to chemotherapy. PSTT accounts for 0.2% of GTD, with a morbidity of around 1/100,000 per delivery. According to studies published in recent years, there are subtle differences in incidence among different regions around the world (4–13). The clinical features of PSTT are usually benign, but a few of them can relapse and metastasize, demonstrating the malignant biological behavior. The early diagnosis of PSTT remains uncertain, and it is difficult to distinguish the benign and malignant forms of PSTT in the early stage (14). The present obstacles in managing PSTT include malignancy prediction, fertility preservation, recurrence and chemotherapy resistance, and identification of possible treatment targets. We discussed the updates concerning these problems and summarized them to three main aspects: mechanism, clinical presentation, and treatment and prognosis.

## Mechanism

### Pathogenesis

**Figure 1:**
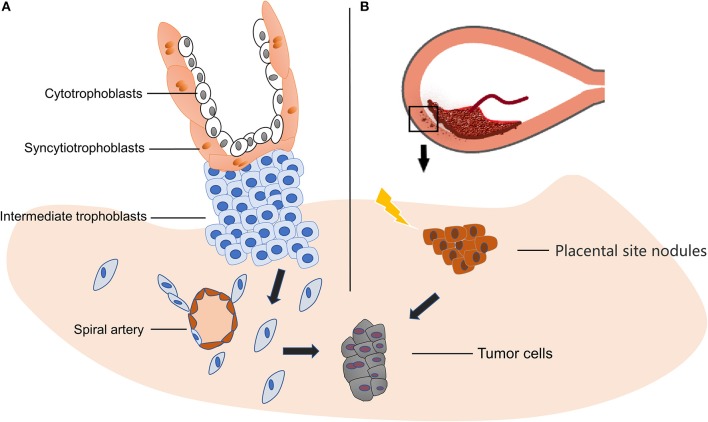
Origin of PSTT. **(A)** PSTT originates from extravillous trophoblasts, and then acquires the abilities of proliferation and migration. Afterwards, those cells migrate away from placenta and invade the decidual artery and uterine spiral artery to remodel the blood vessels which in turn provide nutrition for the embryo. The disruption of this well-regulated invasion process may lead to PSTT. **(B)** During delivery, placenta detaches from decidual, leaving small nodules and form placental site nodules in the myometrium. In the process of reabsorbing, some adverse trigger or stimuli may cause atypical mitosis and result in neoplasm.

Gestational trophoblastic neoplasia (GTN) includes hydatidiform mole, choriocarcinoma, PSTT and epithelial trophoblastic tumor (ETT). PSTT originates from placental site intermediate trophoblasts, which refer to the cells at the distal villus that attach to the endometrium and become dispersed and independent cell lines, and then acquire the abilities of proliferation and migration (Figure 1A) (15). In normal pregnancy, those cells migrate away from placenta and invade the decidual artery and uterine spiral artery to remodel the blood vessels, which in turn provide nutrition for the embryo (15, 16). These features of extravillous trophoblast are similar to those of tumor cells, and facilitate successful placental implantation. The above process is subject to strict temporal and spatial regulation in normal placentation (17). It is considered that PSTT is caused by hyperplasia of intermediate trophoblast, while hydatidiform mole and choriocarcinoma are results of abnormal or malignant proliferation of syncytiotrophoblast and cytotrophoblast (18, 19). Others propose that PSTT forms during the process of placenta detaching from the uterus, and the small nodules of the placental tissue remaining in the myometrium, and being reabsorbed over time. During this complex process, cell mitosis increases and becomes either atypical placental nodules or PSTT (Figure 1B) (18). The occurrence of PSTT is closely related to the change of the invasive ability of trophoblasts, a process involving many cytokines, extracellular matrix components, and enzymes.

### Molecular Mechanism

**Figure 2:**
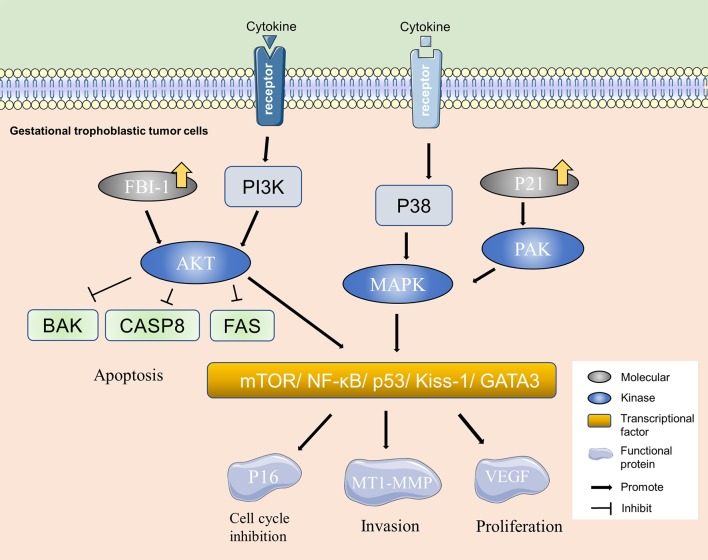
Molecular mechanism of PSTT. PI3K/AKT and MAPK are important molecular pathways in PSTT. Over-expression of molecules, such as FBI-1 and P21 can activate kinases, such as PAK and AKT in gestational trophoblastic tumor cells and consequently cause changes in the adhesion-associated and cell cycle regulation proteins, resulting in alterations of biological behaviors including invasion and proliferation of PSTT.

The alterations of intracellular signaling pathways, intercellular information transmission, and extracellular matrix contribute to the occurrence of tumor. Studies have indicated that ERK, MAPK, mTOR signaling pathway, transcription factor NF-κB, Kiss-1, and GATA3 may play key roles in the invasion and metastasis of PSTT (Figure 2) (20–22). Adhesion molecules mediating cell-to-cell and cell-to-matrix communication can cause metastasis and local inflammation. For example, integrin-α5β1, which expresses on the cell surface and belongs to the adhesion molecule family, can interact with extracellular matrix components and is capable of fixing the cytoskeleton. Studies have found that P-selectin and integrin-α5β1 are related to the occurrence, development, invasion and metastasis of PSTT (23). Other molecules, such as P65, CD44v6, CD146, P21, FBI-1, and E-cadherin, are reported to play a similar role (21, 24, 25). In addition, the destruction of extracellular matrix components facilitates cancer cell invasion and metastasis. Heparanase (HPA) can promote tumor metastasis by degrading heparinase sulfate proteoglycan (HSPG) in extracellular matrix (26). Normally, the expression of HPA is limited to placenta and immune organs (23). It is reported that the expression of HPA is elevated in invasive malignant tumors, such as breast cancer, esophageal cancer, rectal cancer, and bladder cancer (20), and is found to be expressed in extravillous trophblasts (27). Researchers have speculated that HPA is related to the invasion of PSTT. Furthermore, it is implied that matrix metalloproteinase (MMP), tissue inhibitor of metalloproteinase (TIMP), and other molecules in the matrix may promote PSTT metastasis (28).

### Genetic Mechanism

Several types of GTD are characterized by distinct genetic modes (29). There are various hypotheses about the underlying genetic mechanism of PSTT. A study analyzed the status of X chromosome inactivation of PSTT cases, and found that PSTT present a unique genetic mode requiring the presence of a paternal X (Xp) chromosome and PSTT are derived from the extraembryonic tissue of an antecedent female conceptus (30). Duplication of Xp chromosome is considered to cause abnormal genetic overdosing and plays a role in trophoblastic proliferation (30). Evidences have shown that X chromosome-linked genetically imprinted genes regulate extraembryonic tissues (31, 32). X chromosome inactivation is crucial to the development of normal extraembryonic tissues (33). It is believed that uniparental gene expression can lead to cancer predisposition. The hypothesis is that Xp harbors a dominant oncogene or tumorigenesis results from abnormal dosage of functional X chromosomes. Possible oncogenes include *Esx1, Pem, MYCL2*, and *IAP* (32–35). Using short sequence analysis of X chromosome, Zhao et al. confirmed the importance of maternal X chromosome in the occurrence of PSTT (36).

*PHLDA2* (platelet-leukocyte C kinase substrate analog family A-2) is a paternally imprinted gene while expressed maternally, and is located at 11p15.5 which belongs to a known tumor suppressor gene region (37). The PHLDA2 protein is exclusively expressed in extravillous trophoblasts. Studies have found that PHLDA2 increased apoptosis-associated proteins and decreased synthesis of cyclin and cyclin-dependent kinase, thus inducing apoptosis of trophoblasts and reducing the proliferation ability of trophoblasts (37). Genomic hybridization studies have observed regional chromosomal gains involving 21q in PSTT cases, suggesting that chromosomal gains involving 21q may be associated with PSTT pathogenesis (38). We summarized major findings from current genetic studies in Table 1. Future studies are needed to further elucidate the involvement of these genes in PSTT.

**Table 1:** Major findings from genetics studies of PSTT.

**References**	**Sample size (*n*)**	**Method**	**Main findings**
Fisher et al. (39)	2	(1) Y chromosome-specific probe (2) Minisatellite probes to identify highly polymorphic restriction fragment length polymorphisms (RFLPs)	PSTT may originate from both normal term pregnancy and complete hydatidiform mole; no Y chromosome-specific sequences were identified.
Fukunag et al. (40)	3	(1) Immunohistochemistry (2) Flow cytometry	PSTT have an exclusively diploid DNA content.
Hui et al. (30)	26	(1) Sex chromosome analysis (2) PCR-based assay of the androgen receptor (AR) (3) Methylation status analyses	Development of PSTT involves a functional paternal X chromosome.
Oldt et al. (41)	23	(1) Polymerase chain reaction (PCR) (2) Single nucleotide polymorphism (SNP) PCR Assay (3) Mutational analysis of K-ras	Y genetic component was presented in 12 of the 23 (52%) PSTT cases; none of the PSTT cases showed mutation in either codon 12 or 13 of K-ras.
Xue et al. (42)	2	(1) Comparative genomic hybridization (CGH) (2) Chromosome *in situ* hybridization (CISH)	Malignant behavior of PSTT may be not related to the DNA copy number changes.
Hui et al. (38)	4	Comparative genomic hybridization (CGH)	Chromosomal gain involving chromosomal 21q in PSTT.
Hui et al. (43)	20	(1) Polymerase chain reaction (PCR) (2) DNA genotypic analysis (3) DNA hybridization (4) X chromosome polymorphism analysis	The presence of a paternal X chromosome was detected; no Y chromosomal element was found.
Dotto and Hui (44)	20	DNA genotypic analysis	No genetic association between PSTT and exaggerated placental site reaction.
Zhao et al. (36)	33	Molecular genotyping	Antecedent pregnancy of most PSTT cases were female pregnancies.

### Immunological Mechanism

Tumor cells can survive immunological surveillance, escape immunological elimination, and sometimes cause immunological tolerance. The role of immune system in the occurrence of gestational trophoblastic diseases has attracted many attentions in recent years. Immune response disorder on maternal-fetal interface may be related to abnormal reproductive disease. HLA-G is a non-classical class I histocompatibility complex expressed in EVT, which can protect cells from being killed by natural killer (NK) cells and CD40T cells, and maintaining maternal and fetal immunological tolerance. Trophoblasts can up-regulate the expression of HLA-G, which makes it conducive to abnormal proliferation, infiltration, and metastasis of trophoblasts (45). Th1/Th2 and Th17/Treg balance are also closely related to placenta formation and pregnancy maintenance. The changes of Th1/Th2 and Th17/Treg balance in PSTT have not been reported, and should be examined comprehensively (45, 46).

## Clinical Presentation

### Clinical Characteristics

PSTT often occurs in women of childbearing age, with an average age of 32 years. The occurrence of PSTT can follow term pregnancy, premature delivery, hydatidiform mole, and choriocarcinoma, with an interval between the occurrence and previous pregnancy ranging from months to several years. Most PSTT develop within 1 year after the antecedent pregnancy. Tumors of early stage are confined to the uterus and grow slowly. The main symptoms of PSTT include colporrhagia and amenorrhea (2, 13, 47–49). Compared to other trophoblastic tumors, the human chorionic gonadotropin (hCG) serum level is slightly elevated in most PSTT patients. In rare cases, the serum level of hCG can reach even 100,000 IU/L (50), which may be a result of tumors mixing with choriocarcinoma tissues. Although the metastasis tendency of PSTT is lower than that of choriocarcinoma, it occurs occasionally. The main sites of metastasis include lung and the central nervous system (13, 51, 52). Other symptoms, such as hemoptysis, nephrotic symptoms and abdominal mass will appear while the tumor progresses or metastasizes. Very few cases are associated with nephropathy, such as proteinuria and thrombotic microangiopathy (53, 54). One study reported a PSTT case coexisted with paraneoplastic nodular regenerative hyperplasia of the liver (55). Some scholars believe that these symptoms may be caused by paracancerous syndrome which might be induced by the immune response to cancer cells or humoral factors. These paraneoplastic disorders usually resolve after hysterectomy and/or chemotherapy. Table 2 summarizes atypical presentations of PSTT.

**Table 2:** Atypical presentations of PSTT patients.

**References**	**Age**	**Symptoms**	**Antecedent pregnancy**	**Treatment**
Dumas et al. (55)	37-years-old	Paraneoplastic nodular regenerative hyperplasia of the liver	Full-term delivery	Hysterectomy EMA/EP
Sawamura et al. (54)	32-years -old	Thrombotic microangiopathy-like glomerular lesion	Full-term delivery	Hysterectomy
Xiao et al. (53)	31-years -old	Lupus nephritis	Full-term delivery	Hysterectomy with a bilateral salpingo-oophorectomy
Batra et al. (56)	28-years -old	Membranous glomerulopathy	Medical termination of pregnancy	Hysterectomy
Brewer et al. (57)	42-years -old	Erythrocytosis	Full-term delivery of a female infant	Hysterectomy

*EMA/EP, etoposide, methotrexate, actinomycin-D/etoposide, cisplatin*.

### Pathological/Histopathological Features and Differential Diagnosis

**Figure 3:**
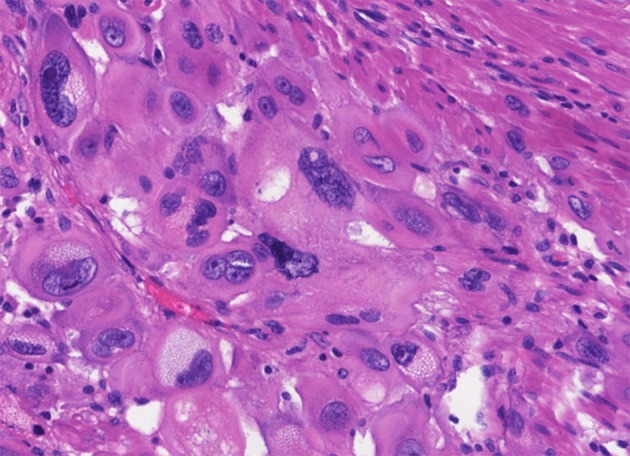
Histopathological features of PSTT. Tumor cells present monomorphic population of large polyhedral cells with irregular hyperchromatic nuclei, which are at different stages of mitosis. Besides, there is eosinophilic or transparent substance in the cytoplasm that could be large amount of fibrin. Tumor cells grow like nest or bands into myometrium, with a handful of bleeding foci and mild inflammation or necrosis.

Different trophoblastic diseases have distinct origins and pathological manifestations. In PSTT, the lesion is generally located at the placenta implantation site, and is massive or polypoid, protruding uterine cavity or infiltrating the uterus. Hemorrhagic focus can be found in PSTT. There is hardly normal villus structure under microscopic observation (58, 59). In addition, there are abundant intermediate trophoblasts, but no cytotrophoblasts or syncytiotrophoblasts (58). PSTT tumor cells infiltrate into the uterine myometrium in a strip-like manner, revealing the invasive ability of the intermediate trophoblasts at the implantation site. PSTT invades deep myometrium of the uterus and extends to serosa in some cases (58). The mitotic figures of PSTT vary. It can be observed that the nuclei are at different stages of mitosis. Tumor cells present monomorphic population of large polyhedral cells with irregular hyperchromatic nuclei. Besides, there is eosinophilic or transparent substance in the cytoplasm that could be large amount of fibrin (52, 60). Despite evident infiltration, the artery walls are usually intact, with a handful of bleeding foci and mild inflammation or necrosis (Figure 3). Immunohistochemistry analyses have shown that the expression of prolactin (hPL), cytokeratin (CK), melanoma adhesion molecule (Mel-CAM), Cyclin E and CD146 is diffuse positive in the cytoplasm of PSTT tumor cells, while the expression of hCG is locally positive and Vimentin is negative (60–63). It has been shown that inhibiting, epithelial membrane antigen (EMA) and placental alkaline phosphatase (PLAP) are focally positive (64). The expression of Ki67, a type of nuclear antigen, is positively correlated to the proliferation ability of tumor tissue. In PSTT, Ki67-positive cells could reach 15%, while in choriocarcinoma it may surge to 60–70% (61).

Comparatively, ETT arises from chorionic-type intermediate trophoblast cells. For ETT, the lesions locate at decidua outside the implantation site, and can be found in the uterine body, fundus and cervical canal. ETT lesions are generally solid or cystic, with certain degrees of hemorrhage and necrosis. Tumor cells display a nested or bulk pattern of growth, similar to the biological behavior of smooth chorion under microscopic observation. There are characteristic geographic changes, referring to necrosis and eosinophilic vitreous matrix around the tumor cells (2). CK18 and P63 present diffuse positive immunoreactivity while hPL is negative or focally positive in the cytoplasm (65). CD146 is negative or locally positive but PLAP, E-cadherin and EGFR are positive on the membrane (66). ETT can be differentiated from PSTT by the expression of hPL and P63 (61). Previous studies have compared the expression of P40 and P63 in PSTT and ETT, and suggested that P40 is an equivalent immunohistochemical marker of ETT (62).

Choriocarcinoma originates from choriotrophoblasts, and the lesions appear in the myometrium of the uterus. Choriocarcinoma can also protrude into the uterine cavity or perforate the serosa. The choriocarcinoma lesions feature significant necrosis and abundant blood supply (67). Microscopically, there are typical cytotrophoblasts and syncytiotrophoblasts with mitotic figure >10/10 HP. Choriocarcinoma has significant vascular infiltration, accompanied by massive hemorrhage and necrosis. In choriocarcinoma, hCG shows strong positive immunoreactivity with the presence of rich cytotrophoblasts alternate with syncytiotrophoblasts, and hPL is focally positive or negative (67). There are a couple of points to help differentiate choriocarcinoma diagnosis from PSTT: Ki67-positive cells exceeding 50% and elevated serum beta-hCG level (67). A small number of studies also looked at other possible markers, such as pregnancy-associated major basic protein (pMBP), which has been shown to stain positively in large exaggerated placenta sites and PSTT cells (63). Table 3 summarizes the different expression levels of above discussed makers. Because of the chemoresistance of PSTT, it is important to establish biomarkers to aid diagnosis and stratification. It is found that glypican-3(GPC3), carcinoembryonic antigen-related cellular adhesion molecule 1 (CEACAM1), GATA binding protein 3 (GATA3), and HLA-G were positively expressed in PSTT (68–71), while bcl-2 and spalt-like transcription factor 4 (SALL4) were negatively expressed in PSTT (69, 70). Among these markers, SALL4 and bcl-2 are modestly positive in choriocarcinoma (69, 70), and HLA-G is exclusively positive in intermediate trophoblasts (71). Table 4 summarized studies evaluating differential diagnoses and potential diagnostic biomarkers of PSTT.

**Table 3:** Summary of the expression patterns of different markers in three types of gestational trophoblastic neoplasia (GTN) tissues.

	**PSTT**	**ETT**	**Choriocarcinoma**
CK	+++	+++	+++
HPL	+++	–/+	–/+
HCG	–/+	–/+	+++
CD146	+++	–/+	–/+
Ki67	+	+	+++
EMA	++	+++	NA
P63	–	+++	–/+
Vimentin	–	+	–

–*, 0–5%; +, 5–30%; ++, 31–60%; +++, >60% expression*.

*ETT, epithelial trophoblastic tumor; NA, Not available; PSTT, placental site trophoblastic tumor*.

**Table 4:** Differential diagnosis and potential diagnostic biomarkers of PSTT.

**Biomarker**	**Sample size (*n*)**	**Staining results (*n*)**	**Positive rate**
GPC3 (68)	15	3(–), 3(+), 6(++), 3(+++)	80% (12/15)
p53 (69)	12	5(–), 5(+), 2(++)	58.3% (7/12)
CEACAM1 (69)	12	1(–), 11(+)	91.7% (11/12)
pCEA (69)	12	6(–), 1(+), 5(++)	60% (6/12)
bcl-2 (69)	12	12(–)	0
SALL4 (70)	9	9(–)	0
GATA-3 (22)	6	NA	71%
HLA-G (71)	14	0(–)	100%

–*, no immunoreactivity; +, 1–25% of positive cells; ++, 26–50% of positive cells; +++, 51–75% of positive cells; ++++, 76–100% of positive cells*.

*CEA, carcinoembryonic antigen; CEACAM1, carcinoembryonic antigen-related cellular adhesion molecule 1; GATA-3, GATA binding protein 3; GPC3, glypican-3; HLA-G, Human leukocyte antigen, class I, G; NA, not available; pCEA, polyclonal antibodies; SALL4, spalt-like transcription factor 4*.

Taken together, different trophoblastic diseases have distinct origins and pathological manifestations. Although the immunochemistry markers as well as serum beta-hCG level contribute to the differential diagnosis of GTN, it remains ambiguous for some patients. It is helpful to develop more non-invasive diagnostic markers to aid accurate diagnosis.

### Liquid Biopsy

In recent years, liquid biopsy, which contains circulating tumor cells (CTCs), circulating tumor DNA (ctDNA), circulating tumor RNA (ctRNA) and extracellular vesicles (EVs), has become a useful tool in cancer diagnosis. Studies have shown that solid tumors release enough ctDNA into the bloodstream to be detected. The analysis of ctDNA can help with molecular genotyping, mutational analysis, diagnosis of cancer, and surveillance after treatment. Previous study has shown that it is feasible to extract cell free DNA (cfDNA) to utilize as “liquid biopsy” in patients without histopathological diagnosis (72). There is huge potential of liquid biopsy in PSTT. First of all, liquid biopsy can be used for diagnosis and differential diagnosis. In combination with molecular technology, such as short tandem repeat (STR), single nucletide polymorphism (SNP) and amplification refractory mutation system-PCR (ARMS-PCR), liquid biopsy can detect paternal or maternal alleles to aid the diagnosis of GTN. Given the presence of non-maternal DNA in GTN tumors, the characteristics of these tumor cells in circulation may be easily detected. Then, the amount of ctDNA may reflect tumor burden and has been found to be associated with HCG in invasive molar disease (72–74). Besides, liquid biopsy can be used for post-operative monitoring of recurrence and metastasis. When histology is unavailable or minimal metastasis occurs which cannot be detected by imaging, ctDNA may be used to aid early diagnosis with advantages of painlessness, low risk and low cost (75). In addition, ctDNA may provide unique genetic information that can help developing personalized treatment. Furthermore, the level of ctDNA may be affected by chemotherapy, therefore ctDNA may be used to detect drug resistance mutations (75).

### Imaging Features and Differential Diagnosis

**Figure 4:**
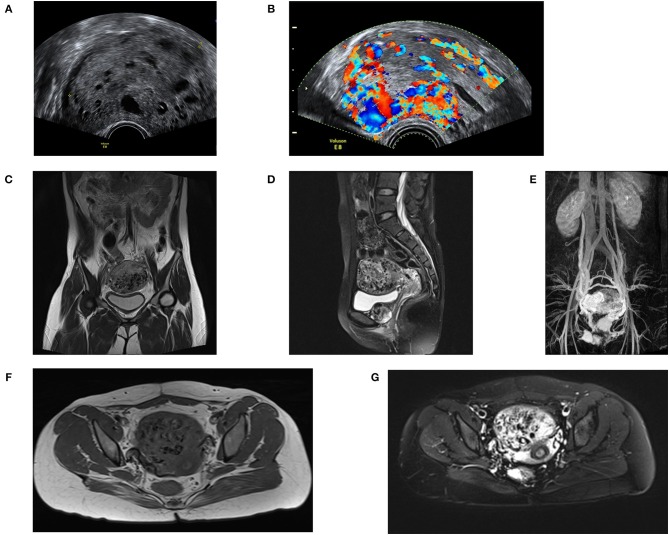
Imaging features of PSTT. **(A)** Ultrasound feature of PSTT: the mass shows as a mixed mass with intact capsule and presents as echogenic bulk. **(B)** Ultrasound feature of PSTT: formation of arteriovenous fistula can be observed. **(C,D)** MRI imaging of sagittal position: PSTT presents as uterine dilatation with short T1 and long T2 signals. **(E,F)** MRI imaging of axial position. **(G)** Enhancement imaging: the parauterine artery can be observed.

Imaging manifestation including ultrasound, computer tomography (CT) and PET-CT can assist the diagnosis of PSTT. Detailed gynecologic ultrasonography is used for ultrasound-guided dilation and curettag (D&C) or hysteroscopically guided biopsy to help with the diagnosis and preoperative staging (76). In combination with clinical history and hCG levels, ultrasonography is a first-line imaging method for GTN diagnosis because of its convenience and economy (76, 77). Both transabdominal and transvaginal ultrasound are performed. The sonographic findings can show changes in size, morphology, echo, surrounding lymph nodes and the relationship between the uterus, and the surrounding organs. It is important to assess the extent of the lesion and the choice of treatment. PSTT can be classified according to the imaging findings of the lesion location. There are three types of PSTT based on transvaginal ultrasound: most of the tumors protruding into the uterine cavity (Type I), the lesion is located in the uterine cavity and part of the myometrium (Type II), and the whole lesion is in the myometrium (Type III). Most lesions are located at the uterine corpus, and very few cases reported lesions in the uterus cervix or pelvic wall (78–80). According to the literature, the size of lesions ranges from 7 to 62 mm (81, 82). The lesions are solid or cystic or mixed with solid capsules. About one-half of the lesions appear to be hypoechoic or echogenic (77). There is no obvious boundary between the lesion and the surrounding tissue. Those cystic and mixed cystic lesions can be misdiagnosed as choriocarcinoma or hydatidiform mole. The blood flow signal of the PSTT lesion is slow, and the blood flow resistance index is low (76, 77, 83). The hemodynamic parameters of PSTT tumor vessels show that the Doppler signal is distributed in the lesion and the blood flow signal is rich, which may be caused by vasodilation or formation of arteriovenous fistula (Figures 4A,B) (84). PSTT can be divided into vessel abundant and relatively low blood vessel types detected by the Doppler signal. D&C should be avoided in the vessel abundant type. Compared to PSTT, the ultrasound of ETT is characterized by an echogenic mass located in the myometrium of the uterus, with abundant blood flow signals (83). Geographic necrosis is usually presented in ETT.

Although ultrasound performance is not considered as a diagnostic criterion for PSTT according to the International Federation of Gynecology and Obstetrics (FIGO), it is believed that solid lesions combined with low blood flow signals and low levels of serum βHCG constitute the diagnostic conditions of PSTT (85). In addition to the diagnostic value, ultrasound is a powerful imaging method in subsequent treatment and monitoring processes. Some researchers have suggested that the uterine artery pulsation index (UAPI) <1 can be used as a predictor of chemoresistance risk. Moreover, color Doppler ultrasound is a highly sensitive method for observing residual lesions (86).

MRI is also an imaging method for assessing the condition of PSTT. An abnormal heterogeneous signal changes can be observed in the wall of uterus in the T2-weighted image. In reported cases, MRI usually presents as uterine dilatation with short T1 and long T2 signals (Figures 4C,D,F,G) (77), and enhancement of vascular empty effect in myometrium and bilateral para-uterine tissue. Enhanced heterogeneous tumor and parauterine tissue can be observed after administration of contrast material (Figure 4E). For patients whose tumors cannot be detected by ultrasound, MRI can be used for localization (77, 87).

PET is not considered as regular imaging tools. It has not been widely used in assessing PSTT and other types of GTD cases (88). However, it is reported that PET is highly consistent with ultrasound, chest X-ray, and CT in the staging of GTN, which is not superior to traditional imaging methods, but nevertheless plays an important role in the diagnostic evaluation of high-risk patients. Therefore, it is believed that there is great potential of PET in assessing the range of lesions as well as metastases (88–90). In addition, there is high sensitivity of PET in detecting metastatic lesions (91). F-FDG PET can observe active tumor lesions and hence can be used to monitor PSTT recurrence and chemoresistance.

A study from the United States suggested implementing MRI to the head and pelvis, and CT to the chest and abdominal, and Doppler ultrasonography to the pelvis (77). The location and size of the lesion will be more comprehensive and precise after integrating the results from CT, ultrasound and MRI. The value of FDG-PET in the initial staging is still unclear, and clinicians tend to reserve it for selected cases with unsure lesions or to determine the resection site (91, 92). Considering the unreliability of hCG monitoring, there are great expectations for imaging tools in prognosis surveillance.

## Treatment and Prognosis

### Treatment

#### Surgery

Generally, there are two treatment strategies based on patient evaluation: simple hysterectomy and systemic therapy (93–95). The consensuses are that patients of FIGO stage I can be cured by simple hysterectomy with or without pelvic lymph node biopsy (48). In a 17-year clinical trial of PSTT, of all the patients underwent surgery, 5 out of 13 patients relapsed and the recurrence rate was up to 50% (96). This high rate of recurrence reveals that the management of metastatic lesions is crucial in surgery. It is now generally accepted that lymphatic metastasis is more likely to occur in PSTT compared with choriocarcinoma (97). The retroperitoneal lymph nodes, especially the paraaortic lymph nodes, are the most frequent sites of lymphatic metastasis. Some researchers believed that paraaortic lymph nodes were an important part of lymphatic communication and metastasis between PSTT tumor and pelvic lymphoid tissue. They observed that lymph node metastasis existed in 39.1% of the 24 patients in the study (97). For patients with stage II to IV PSTT, lymph node biopsy should be carried out to evaluate the status of lymphatic metastasis, and provide guidance of whether to perform lymphadenectomy. Multidisciplinary management is important in treating PSTT. It remains controversial in terms of whether to perform oophrectomy. Individual study reported metastasis of PSTT to the ovary (98), which illustrates the possibility of ovarian metastasis and the necessity of oophrectomy in the operation. However, it requires comprehensive evaluation of patient conditions including age, staging, metastasis, basic condition, and fertility requirements before deciding whether to perform oophrectomy. Besides, researchers suggest ovarian retention during hysterectomy should take consideration of family history of ovarian diseases. For PSTT patients with a strong family history of ovarian diseases, bilateral oophrectomy should be seriously considered to reduce the risk of ovarian metastasis.

#### Chemotherapy

Systemic therapy is considered according to risk factors after the surgery. The current treatment guidelines of PSTT vary (Table 5) (46, 99–101). Noticeably, WHO prognostic index score for GTN does not apply to PSTT. Various researches have reported several criteria related to poor prognosis of PSTT, including interval between antecedent pregnancy >2 years, deep infiltration, necrosis, mitotic index >5/10 under microscope. Patients with high risk are recommended to use multi-drug combined chemotherapy. For patients of FIGO stage II–IV, hysterectomy followed by adjuvant chemotherapy is an acceptable choice (52). For patients with metastasis, it is believed that targeted surgery and high-dose platinum-containing chemotherapy containing platinum or etoposide are advisable treatment options. Chemotherapy regimens with reported better response include EMA/EP (etoposide, methotrexate, actinomycin-D/etoposide, cisplatin), EMA/CO (etoposide, methotrexate, actinomycin-D/vincristine, cyclophosphamide), and TE/TP (taxol, etoposide/taxol, cisplatin) (102). Studies have shown that EMA/EP lead to a higher remission rate; however, it has high toxicity and possible subsequent hematological side effects, such as neutropenia (103). TP and TE regimens are also used in the clinic. For relapsed patients, EMA/EP and TP show more chemosensitivity than other regimens. Therefore, it is the first line treatment choice especially for refractory or relapsed PSTT patients. In addition, salvage chemotherapy regimens including BEP (bleomycin/etoposide/platinum) and ICE (ifosfamide/cisplatin/etoposide) are used in chemotherapy-resistance patients (104).

**Table 5:** Recommended treatment of PSTT in published guidelines.

**Association**	**First-line option**	**Alternative regime**	**Risk factors of poor prognosis**
NCCN (99)	EP/EMA	TE/TP VIP BEP	(1) Interval between antecedent pregnancy >2 years; (2) Myometrial infiltration; (3) >5 mitotic figures per 10 HP; (4) Necrosis.
ISSTD (100)	EP/EMA	EMA/CO TE/TP M-EA	(1) Interval >4 years from the causative/last known pregnancy; (2) FIGO stage; (3) Number and site of metastasis; (4) hCG level and mitotic index; (5) Lympho-vascular invasion and/or spread outside the uterus.
Chinese consensus (46)	EMA/CO	BEP VIP ICE	(1) >5 mitotic figures per 10 HPF; (2) Interval between antecedent pregnancy >2 years; (3) Extrauterine metastasis; (4) Higher stage; (5) Course of disease >4 years; (6) Transparent cytoplasm.
DGGG (101) OEGGG (101) SGGG (101)	EMA/CO or EP/EMA	BEP	(1) Tumor growth extending beyond the uterus; (2) High-grade nuclear and cellular polymorphism; (3) Extensive coagulative necrosis; (4) Destructive growth; (5) Deep myometrial infiltration; (6) Evidence of trophoblastic cells with clear cytoplasm; (7) > 5 mitotic figures per 10 HPF; (8) High proliferative activity (Ki-67 labeling index) of more than 50%.

*BEP, bleomycin etoposide cisplatin; DGGG, German Society of Gynecology and Obstetrics; EMA/CO, etoposide methotrexate actinomycin-D/cyclophosphamide vincristine; EP/EMA, etoposide cisplatin/etoposide methotrexate actinomycin- D; ICE, ifosfamide cisplatin etoposide; ISSED, International Society for the Study of Trophoblastic Disease; M-EA, methotrexate-etoposide dactinomycin; NA, not available; OEGGG, Austrian Society of Gynecology and Obstetrics; SGGG, Swiss Society of Gynecology and Obstetrics; TE, paclitaxel etoposide; TP, paclitaxel cisplatin; VIP, vindesine ifosfamide cisplatin*.

It is reported that high-dose chemotherapy regimen containing platinum significantly increased overall survival in patients with high-risk GTD, and 5 of 11 high-risk PSTT patients receiving the treatment were in remission (105). In UK, researchers adopted an 8–12 weeks EP-EMA regime based on patient tolerance, after which stem cell mobilization is initiated, then several courses of high dose chemotherapy are applied. Following the above processes, autologous stem cell transplant support sequential therapy is implemented (106). The authors proposed that early initiation of high dose chemotherapy and peripheral blood stem cell support therapy in high-risk PSTT patients with an interval between antecedent pregnancy >2 years, and in younger patients with low HCG levels are more effective (106, 107). The disadvantages of high-dose chemotherapy are toxic side effects and induced premature ovarian failure (POF) which will affect the patient's future fertility. Chemotherapy may lead to decreased follicular cells, thus decreased ovarian reserve, and premature ovarian failure. Anti-Müllerian hormone (AMH) is a valuable marker that reflects the function of particles and sertoli cells. It can be used for assessing female ovarian reserve and monitoring menopausal process. Researchers have observed that serum AMH levels are significantly decreased after using etoposide (VP16) in patients with GTN, and proposed that monitoring AMH levels during chemotherapy is beneficial for ovarian protection (108).

In general, PSTT patients are prone to chemotherapy resistance and should be treated with appropriate surgical treatment in time. In addition, chemotherapy response for each patient should be determined timely so as to avoid low reactivity. For patients with metastasis, comprehensive treatment strategies, such as high-dose multidrug combination chemotherapy, surgery, imaging evaluation, and the use of colony stimulating factor (G-CSF) are implemented. To the best of our knowledge, there is currently no literature on how to evaluate chemotherapeutic reactivity of trophoblastic tumors. Treatment experiences of other types of tumor have shown that measurement of chemotherapy efficacy can base on clinical benefit response (CBR), which is tumor-related symptoms after chemotherapy improvement; drug susceptibility test; imaging evaluation including CT, MRI, radionuclide scanning, and angiography; tumor size; tumor blood supply; and tumor density changes (99). Besides, currently there is no monitoring of drug resistance genes in PSTT patients, which could also be a direction for future research to explore.

#### Fertility Preservation

Since most patients with PSTT are young women, the preservation of fertility during chemotherapy is a critical issue. At present, the means of conservation is mainly based on surgery and chemotherapy. The choice of surgical methods includes abdominal resection, laparoscopic resection, and hysteroscopic resection (6, 109, 110). There is also report of using a modified Strausmann procedure (MSP) on suitable candidates (111). Some researchers have observed significant effects of arterial infusion of chemotherapy drugs, which significantly increased uterine preservation therapy (112).

The success rate of fertility preservation treatment is relatively high in young women. However, a small number of patients who went through fertility preservation therapy eventually underwent hysterectomy, probably due to incomplete removal of residual lesions. A study conducted in a single hospital reported that about 21% of patients who preserved uterus in surgery successfully preserved fertility (6). Another study reported that when trying to perform local resection of tumors, five out of six PSTT patients finally underwent total hysterectomy because of failure to completely remove hidden lesions (111). The obstacle of fertility-preserving treatment lies in the establishment of criteria to distinguish patients that are suitable to preserve fertility. Scholars have proposed appropriate conditions for fertility preservation, those factors include stage I, >35 years old, a strong fertility requirement, acceptable chemotherapy responsiveness, no malignant prognostic factors, such as deep myometrium infiltration (110, 113). More data are needed to confirm those findings.

#### Targeted Therapy

Developing new strategy for treatment of PSTT is of great significance. Recently, certain treatment strategies, such as targeted therapy for vascular growth factors and immunological checkpoints, as well as the application of mifepristone have drawn attention (114). Targeted therapy can increase the efficacy while reducing side effects because they selectively target specific pathways. Standard chemotherapy affects most proliferating cells, while target-based treatment is designed to inactivate molecular pathways, such as PI3K and MAPK signaling pathways that are essential for tumor-cell growth and survival (115). Studies have found increased expression of vascular endothelial growth factor (VEGF) and TGFβ3 in PSTT tissues (116, 117). VEGF and TGFβ3 are both growth factors that play an important role in angiogenesis, embryo implantation and placenta formation. TGFβ3 has many regulatory effects on trophoblasts, such as inhibiting proliferation and invasiveness. Importantly, the time sequence of TGFβ3 expression is consistent with the regulation of trophoblast invasion throughout pregnancy. It is believed that overexpression of VEGF and TGFβ3 may enhance the invasiveness of trophoblastic tumors and associated with poor prognosis (118). Another vascular growth factor, endocrine gland-derived vascular endothelial growth factor (EG-VEGF), is exclusively expressed in endocrine tissues (ovary, testis, adrenal cortex, and placenta), but has no effect on endothelial cells of other tissues. EG-VEGF can induce phosphorylation of p42/p44 MAP kinases and Akt pathway through receptors PKR1 and PKR2. It is reported that there were differences in the expression of EG-VEGF and PKR1/PKR2 between normal villous tissues and choriocarcinoma cell lines, and that PKR1 was mainly expressed in cytotrophoblasts, while PKR2 was mainly expressed in extravillous trophoblasts and syncytiotrophoblasts (119). Inhibition of PKR2 expression by small interfering RNA demonstrated that EG-VEGF can inhibit EVT invasion by regulating MMP-2 and MMP-9, suggesting that EG-VEGF may be an important factor in the development of trophoblastic tumors (119). Further studies are needed to investigate whether targeted therapy of VEGF, TGFβ3, and EG-VEGF can assist PSTT diagnosis or treatment.

#### Immunotherapy

Immunotherapy has been proven to be successful in treating several types of cancer patients after first-line chemotherapy (120–123). It has been proposed that there is potential of immunotherapy in treating PSTT patients (124). The immunological checkpoints have gained wide attention in recent years. Programmed death 1 (PD1) is a transmembrane receptor expressed on the surface of T cells, B cells, NK cells, as well as antigen presenting cells. After binding to its ligand (PD-L1), PD-1/PD-L1 can inhibit the production of activated T cells, which is involved in the regulation of immunosuppressive function and immune tolerance. Inhibitors against this membrane surface protein can block the binding of PD-1/PD-L1, thereby relieving the immunosuppressive effect and promoting the killing effect of T cells on tumor cells (125, 126). It has been shown that PD-L1 is widely expressed in all trophoblasts except cytotrophoblasts, while PD-L1, B7-H3, and VISTA were positively expressed in PSTT tissue, indicating that PD-L1 blocker may be a potential treatment for PSTT (125, 126). PD-1 blockers (Nivolumab, Pembrolizumab) and PD-L1 blockers (Atezolizumab, Avelumab, Durvalumab) have been used in treatment of cancer (120–123). A recent study has shown that Pembrolizumab is a remarkably effective drug in patients with chemotherapy-resistant gestational tumors (124). Besides, it has been shown that intravenous infusion of Pembrolizumab is well-tolerated with an accepted toxicity profile, which makes it a promising treatment choice (124).

Since most PSTT patients are young women preferring fertility-preserving treatment, which usually is simple local resection, there is elevated risk of recurrence and metastasis. Most PSTT patients develop chemotherapy resistance to platinum-based regime, and PD-1 inhibitors, such as Nivolumab and Pembrolizumab have been used to treat patients with platinum-resistant ovarian cancer patients (121). Therefore, the use of PD-1 antibody might be a life-saving therapy for chemotherapy-resistant patients and improve the prognosis of PSTT. In addition, PD-1/PD-L1 can be used in combination with a CTLA4 inhibitor or an anti-angiogenic targeted drug to enhance the therapeutic effect (127, 128). Because of the memory function of the immune system, once the PD-1 inhibitor works, patients will have a chance to achieve long-term cure, which has already been observed in malignant tumors, such as malignant melanoma (129–131). The prediction of responsiveness to immunotherapy could be based on human lymphocyte antigen class I (HLA-G), microsatellite instability-high (MSI-H), deficient mismatch repair (dMMR), microsatellite detection (MSI), tumor mutation burden (TMB), and tumor infiltrating lymphocytes (122, 124). However, the application of immunotherapy in treating PSTT patients requires further clinical investigation.

Immune checkpoint inhibitors are expected to increase the treatment success rate, while currently there is no research about pregnancy outcomes after using immune checkpoint inhibitors in patients with trophoblastic diseases. Existing literatures have reported several immune-related side effects caused by immune checkpoint inhibitors, such as endocrine system disorders, sexual dysfunction, and damage to the reproductive system (132). Another theory is that inhibition of the PD-1 pathway can reduce immune tolerance of the maternal-fetal interface during pregnancy (133, 134). Whether and/or how these side effects will affect the long-term reproductive function remains to be elucidated in the future.

### Prognosis

The prognosis of most PSTT cases is desirable; however, metastasis or recurrence does occur. Detailed information regarding prognosis has been summarized in a previous review (48). FIGO staging is the main factor affecting PSTT prognosis (6, 10). A retrospective study with 62 PSTT cases reported a 10-years survival rate of 90% for stage I patients, 52% for the stage II patients, 49% for stage III and IV patients (87).

Patients with stage I PSTT usually experience favorable prognosis after total hysterectomy, and the 10-years survival rate can be as high as 100% (112). However, those PSTT patients became infertile due to hysterectomy and may suffer from subsequent psychological and social stress (113). The prognosis of patients with stage II-IV PSTT is relatively poor. Although the progression of PSTT placental trophoblastic tumor is generally slow, it has certain malignant potential. According to the statistics from Britain, the 10-years survival rate of patients with stage II-IV PSTT was around 49% during 1976–2006 (112). The prognosis of patients with recurrent or refractory diseases is worse, with a 5-years survival rate of about 22% (112). A study reviewed and analyzed the prognosis of 88 PSTT cases and reported that the survival rate of patients with stage III–IV PSTT was only 30% (135).

Chemotherapy resistance is a major obstacle of PSTT prognosis. A study from Beijing showed that among all 108 PSTT cases only 7 deaths were observed, which were due to poor chemotherapy response and recurrence (6), indicating early diagnosis and accurate chemotherapy are essential to improved outcome. There is an urgent need for prognostic and predictive biomarkers for patients' stratification. It is suggested that immunoactivity of VEGF and EGFR indicates the efficacy of targeted therapy (97). A study has shown that p53 successfully discriminated confined and metastatic PSTT cases (69). There are also markers, such as MMP and TIMP that could predict biological behaviors including invasive and metastatic abilities of PSTT (28). At present, there are few studies investigating the mechanism of PSTT chemotherapy resistance. A better understanding of the molecular mechanism of PSTT chemotherapy resistance is crucial to develop novel and more effective treatment strategies of PSTT.

## Conclusion

**Figure 5:**
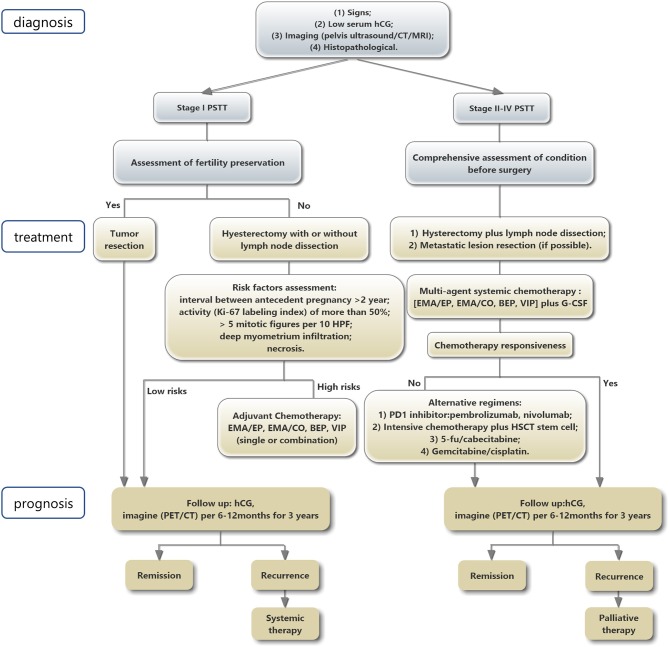
Schematic presentation of PSTT diagnosis, treatment, and prognosis.

To summarize, PSTT is a type of trophoblastic neoplasm with very low incidence and benign characteristics. Similar to many other types of tumors, PSTT may be a result of comprehensive interaction between genetic, immunological, and environmental factors. The prognosis of most PSTT cases is desirable; however, metastasis or recurrence occurs in a few cases. Most PSTT patients are young women hence fertility preservation is an important consideration. Chemotherapy resistance is a main obstacle for PSTT patient prognosis, but the underlying mechanism is still unclear. Table 6 discussed related treatments and survival outcomes of PSTT reported in the recent literature, and Figure 5 summarized the diagnosis, treatment, and prognosis of PSTT patients. Since the disease is very rare, it is important to have a centralized registration system and well-kept data, so as to facilitate PSTT research. Well-conducted multi-center studies with sufficient sample sizes are of great importance to better examine the pathological progress and evaluate the prognosis of PSTT patients. More studies are needed to explore the exact mechanism of PSTT, so as to develop prevention and early detection programs, as well as novel treatment strategies, and finally improve prognosis for PSTT patients.

**Table 6:** Related treatments and survival outcomes of PSTT reported in the recent literature.

**References**	**Sample size (*n*)**	**Stage**	**Treatment (*n*)**	**Chemotherapy (*n*)**	**Survival**	**Prognostic factors**
Zhang et al. (4)	42	Not clear	Local resection (5)	EMA-CO, BEP, EMA-PE	Recurrence: 3 Non-recurrence: 39	ISAP ≧ 2 years, extrauterine metastasis, fertility-sparing surgery
Froeling et al. (106)	125	I: 6 II: 3 III: 4 IV: 4	TAH (49), TAH+BSO (42), TAH+USO (6), fertility preserving (5), other (10)	EP/EMA (21), EMA/CO (18), TE/TP (16), other (22), high-dose chemotherapy (12)	CR: 100 Death: 25	Age, FIGO stage, time since antecedent pregnancy, hCG level, mitotic index
Lee et al. (136)	6	I: 6	Hysterectomy (100%): TAH, SPA-H, RSO, LAVH, LS, BS, ROC, PLND	None	NED	Not reported
Nie et al. (137)	60	I: 60	Surgery	Chemotherapy	Recurrence-free survival rate is 96.7% for surgery alone and 79.1% for surgery + post-operative chemotherapy.	Not reported
Zhao et al. (6)	108	I: 71 II: 4 III: 31 IV: 2	Hysterectomy (85), including hysterectomy alone (19); fertility preservation (23), including fertility preservation alone (3)	Chemotherapy (86)	Mean survival (months) I: 171.3 months II: 43 months III: 123.8 months IV: 9.5 months	Stage, necrosis, deep myometrium involvement, interval between antecedent pregnancy >36 months, prognosis score
Zheng et al. (138)	7	I: 7	LAVH (5), abdominal (1)	Single (3), combined (1), none (3)	NED: 6 Recurrence: 1	Maximum β-hCG, mitotic index
Ozalp et al. (139)	17		Hysterectomy (9)		Remission: 100%	Not reported
Hyman et al. (5)	17	I/II: 8 III/IV: 9	TAH, BSO, LND, including surgery alone	EP/EMA, EMA/CO, methotrexate, BEP	NED: 11 DOD: 6	Stage, interval from antecedent pregnancy, hCG >2,000 Iu/L, age >40
Moutte et al. (7)	15	I: 12 II: 1 III: 0 IV: 2	Hysterectomy (14), including surgery alone (11)	Chemotherapy (4), including chemotherapy alone (1)	Not reported	FIGO stage, a prolonged interval between the antecedent pregnancy, diagnosis of the tumor

*BS, bilateral salpingectomy; BSO, bilateral salpingo-oophorectomy; DOD, died of disease; EMA/CO, etoposide, methotrexate, actinomycin-D alternated with cyclophosphamide, vincristine; EP/EMA, etoposide, cisplatin alternated with etoposide, methotrexate, actinomycin-D; LAVH, laparoscopic assisted vaginal hysterectomy; LS, left salpingectomy; NED, no evidence of disease; PLND, pelvic lymph node dissection; ROC, right ovarian cystectomy; SPA-H, single port assisted laparoscopic hysterectomy; TAH, total abdominal hysterectomy; TE/TP paclitaxel, etoposide alternated with paclitaxel, cisplatin; USO, unilateral salpingo-oophorectomy*.

## Ethics Statement

The study protocol was approved by the Institutional Review Board (IRB) of the Obstetrics and Gynecology Hospital of Fudan University (Reference number: 2016-26; Date of approval: 18 April 2016). The patients gave written informed consent.

## Author Contributions

XF: extensive literature search and drafting. ZW: figures. SZ: extensive literature search. YD: literature search and critical revision. HZ: conception of the work and final version approval. All authors read and approved the final manuscript.

### Conflict of Interest

The authors declare that the research was conducted in the absence of any commercial or financial relationships that could be construed as a potential conflict of interest.
